# Attitudes towards deprescribing and patient-related factors associated with willingness to stop medication among older patients with type 2 diabetes (T2D) in Indonesia: a cross-sectional survey study

**DOI:** 10.1186/s12877-022-03718-9

**Published:** 2023-01-12

**Authors:** Monika Pury Oktora, Cindra Tri Yuniar, Lia Amalia, Rizky Abdulah, Eelko Hak, Petra Denig

**Affiliations:** 1 University Medical Center Groningen (UMCG), Department of Clinical Pharmacy and Pharmacology, University of Groningen, Groningen, The Netherlands; 2 School of Pharmacy, Department of Pharmacology and Clinical Pharmacy, Institut Teknologi Bandung (ITB), Bandung, Indonesia; 3 Faculty of Pharmacy, Department of Pharmacology and Clinical Pharmacy, Universitas Padjadjaran (UNPAD), Bandung, Indonesia; 4 Groningen Research Institute of Pharmacy, Unit of PharmacoTherapy, -Epidemiology and –Economics, University of Groningen, Groningen, The Netherlands

**Keywords:** Deprescribing, Indonesia, Older patients, rPATD, Type 2 diabetes, Survey

## Abstract

**Background:**

Deprescribing of preventive medication is recommended in older patients with polypharmacy, including people with type 2 diabetes (T2D). It seems that many patients in low-middle-income countries are not willing to have their medicines deprescribed. This study aims to assess attitudes of Indonesian patients with T2D towards deprescribing in general and regarding specific cardiometabolic medicines, and factors influencing their willingness to stop medicines.

**Methods:**

Primary care patients with T2D of ≥60 years in Indonesia completed the revised Patients’ Attitudes Towards Deprescribing (rPATD) questionnaire. Attitudes in general and for cardiometabolic medicines were reported descriptively. Proportions of patients willing to stop one or more medicines when recommended by different healthcare professionals were compared with Chi-square test. Multiple regression analysis was used to analyse the influence between patient-related factors and the willingness to stop medicines.

**Results:**

The survey was completed by 196 participants (median age 69 years, 73% female). The percentages willing to stop medicines were 69, 67, and 41%, when the general practitioner (GP), the specialist, or the pharmacist initiates the process (*p*-value < 0.001). Higher perceived burden of medicines (p-value = 0.03) and less concerns about stopping (p-value < 0.001) were associated with a higher willingness to stop medicines if proposed by the GP. Patients using multiple glucose-regulating medicines were less willing to stop (*p*-value = 0.02). Using complementary or alternative medicines was not associated with the willingness to stop. If proposed by their pharmacist, patients without substantial education were more willing to stop than educated patients.

**Conclusions:**

Only two-thirds of older people with T2D in Indonesia were willing to stop one or more of their medicines if the GP or specialist recommended this, and even less when the pharmacist proposed this. Attention should be given to concerns about stopping specific medicines, especially among patients using multiple glucose-lowering medicines, who may be more eligible but were less willing to accept deprescribing.

**Supplementary Information:**

The online version contains supplementary material available at 10.1186/s12877-022-03718-9.

## Introduction

Medication management in older patients with type 2 diabetes (T2D) is challenging, since comorbidities, polypharmacy, and risk of complications are common in those patients [1]. Additionally, treatment goals for older people with diabetes should be individualized considering the time frame of benefit [2]. American and European diabetes guidelines have shifted to a more personalized approach, in which treatment can be deintensified, especially in older patients [3, 4]. Deprescribing is described as the process of reducing or stopping medication to improve patient outcomes in a well-planned process with supervision of healthcare professional [5]. The concept of deprescribing glucose-lowering medicines in people with diabetes is supported, although its implementation in practice appears to be challenging [6]. Most studies, however, have been conducted in the United States of America or European countries.

The process of deprescribing requires interaction between patients and healthcare professionals (HCPs), thus the patient’s involvement is considered an important factor for the success of deprescribing [7]. There seems to be a lot of variation in the percentage of patients willing to have medication stopped in general, ranging from 49 to 98%, which may be influenced by the country, setting or population included [8–10]. Patients may have different attitudes towards deprescribing depending on the specific medicines involved [11, 12]. In particular, the patients’ perceived appropriateness to stop certain cardiometabolic medication may differ between glucose-lowering, blood pressure-lowering and lipid-lowering medicines [11]. There are other factors that may influence the patient’s attitudes towards deprescribing, such as their education level and the number of medicines taken [9, 10]. Previously, it was found that the use of complementary or alternative medicines (CAM) negatively influenced adherence to using prescribed medicines in Indonesian patients with diabetes [13]. It is not known whether this also influences their willingness to stop such medicines. Furthermore, the healthcare professional and the healthcare setting may influence patients’ willingness to have medication deprescribed [9, 10]. It was found that trust in the healthcare professional is important but little is known about the influence of the type of healthcare professional initiating deprescribing on how patients would respond. One study in Croatia found that patients were comfortable with pharmacists’ involvement in deprescribing process, and that they had a positive opinion on their pharmacists’ competencies regarding deprescribing [12]. In addition, patients who perceived more effective communication with their general practitioner or pharmacist were more willing to accept deprescribing [14]. At country level, it was found that patients in low-middle-income countries (LMIC) may be less willing to stop medication compared to high-income countries [9].

No previous study has explored the attitudes of Indonesian older people regarding deprescribing of their medication. Given the rapidly growing number of older patients with T2D in LMICs who are exposed to polypharmacy, further study is needed to gain insight in their willingness and particularly their concerns towards deprescribing. The aims of this study are to assess (1) the attitudes of Indonesian older people with T2D towards deprescribing of medicines, (2) their willingness to stop medicines when recommended by different HCPs, (3) their specific attitudes towards deprescribing of different cardiometabolic medicines, and (4) whether patient-related factors, including the use of CAM and different types of medication, are associated with their willingness to stop medicines.

## Methods

### Study design, setting, and patients recruitment

A cross-sectional survey study was conducted among older outpatients with T2D in Bandung City, West Java Province, Indonesia, from November 2021 to March 2022. Primary care centers in Bandung were selected as sampling sites based on managing at least 40 diabetes patients. Potential participants were recruited by research assistants using convenience sampling among those who visit the sites to collect their medication at that time. The sample was expected to be representative for T2D outpatients living in a large Indonesian city. We conducted an on-site survey using a paper-based questionnaire in Indonesian language. Participants were included who were: (1) aged 60 years and older, (2) received blood glucose-lowering medicines, (3) were literate and able to complete a questionnaire, (4) gave informed consent. The patients completed the survey by themselves, but when needed, participants were helped by the research assistants to fill the information on the number and type of medicines they received. The assistants were pharmacy bachelor graduates who were instructed not to interfere or influence the patients’ answers.

A sample size of 195 participants will provide an estimated margin of error of 7% for assessing the attitudes with a confidence level of 95% assuming that the T2D population is very large [15].

All procedures in this research involving human participants were performed in accordance with the ethical standards of the institutional research committee, and the 1964 Helsinki declaration and its later amendments. Informed consent was obtained from all patients after a full explanation of the aim and procedures of the research. Ethical approval was obtained from the Ethical Committee of Universitas Padjadjaran, Indonesia (No. (191/UN6.KEP/EC/2021). The Checklist for Reporting Of Survey Studies (CROSS) [16] was used to guide reporting (Additional file 1).

### Questionnaire used and outcomes

Patients’ attitudes towards deprescribing were assessed using the revised Patient Attitudes Towards Deprescribing (rPATD) questionnaire [17]. The questionnaire has been validated to measure patients’ attitudes towards their medicines and stopping of medicines. We received permission to use and translate the rPATD questionnaire. We translated the English version of rPATD to Indonesian following the steps as suggested by ISPOR Task Force for Translation and Cultural Adaptation Process for Patient-Reported Outcome Measures [18]. Initial translation to Indonesian language was done by two professional translators, whose first language is Indonesian and who are fluent in English. Next, back-translation to English was done by two professional translators, whose first language is English and who are fluent in Indonesian language. The process of checking for differences and reaching agreement on the translation was performed by two junior and two senior researchers. The Indonesian version of the adapted rPATD questionnaire was piloted among 30 Indonesian people who were prescribed at least two medications to test for clarity and correct understanding of the questions. This pilot resulted in minor language adaptations in four questions. The original rPATD questionnaire, the final translated rPATD, the patient data collection form, and the informed consent form can be found in Additional file 2.

The questionnaire has 22 items, with includes two global statements and four domains each containing five statements. The two global questions refer to overall satisfaction with medicines and willingness to stop one or more of their regular medicines when possible. The four domains include (1) burden of medicines, (2) appropriateness of medicines, (3) concerns about stopping of medicines, and (4) involvement regarding medication management. All statements have five-point Likert-scale answer options, which vary from strongly agree to strongly disagree [17]. The scores can be summed to achieve a total score per domain (strongly agree = 5 to strongly disagree = 1) [17]. Scores for the appropriateness domain need to be reversed. Higher sum scores represent an higher perceived burden of medicines, higher perceived appropriateness of medicines, higher concerns about stopping, and higher involvement regarding medication management.

To investigate attitudes across different cardiometabolic medicines, the rPATD statements for the ‘appropriateness’ and ‘concerns about stopping’ domains were be amended by changing the word ‘my medicines’ to a patient’s medicines that could be eligible for deprescribing: (1) this glucose lowering-medicine (of note, this concerned only sulfonylurea), (2) one or more of my blood-pressure lowering medicines, and (3) one or more of my lipid-lowering medicines, as done in a previous study [11]. The research assistants would add the name of the specific medicines prescribed to the patient on the form before the patient would complete the questionnaire.

To assess the patients’ willingness to stop medicines when recommended by different healthcare professionals, the statement about ‘willingness’ was repeated for the following healthcare professionals: general practitioner, specialist, and pharmacist.

### Patient-related factors

Patient-related factors included as determinants for willingness were age, sex, educational level, number of medicines taken, using more than one glucose-lowering medicines, using lipid-lowering medicines, using blood pressure-lowering medicines, and using CAM. All these data were self-reported. In addition, the sum scores for each of the four rPATD domains were included in these analyses.

### Data analysis

Data entry was conducted by the research assistants and all entered data were checked by one of the researchers using the original paper questionnaires. Descriptive analyses were reported for participant characteristics, for the scores on rPATD global questions (satisfaction, willingness), for the individual statements in four domains (burden, appropriateness, concerns about stopping, and involvement), and for the appropriateness and concerns statements of specific medicines (sulfonylurea, blood pressure-lowering medicines, and lipid-lowering medicines). The Likert-scale answer options were collapsed into ‘agree’, ‘unsure’, and ‘disagree’ to align with previous research [11]. The proportions of patients willing to stop medicines if the general practitioner (GP), specialist, or pharmacist, respectively, proposed this were compared using the Chi-square test.

To test for associations between patient-related factors and willingness to stop medicines, binary logistic regression was used with categories of (1) strongly agree or agree versus (2) unsure, disagree, or strongly disagree for the willingness scores as was done in similar research [19].

First, univariate associations between each of the patient-related factors and the willingness scores were tested. Next, the variables showing a univariate association with a *p*-value < 0.20 were included in a multiple regression analysis. Complete case analyses were performed, excluding those with missing values. A p-value < 0.05 was considered statistically significant for the final model with adjusted Odds Ratio (aOR) and 95% confidence intervals (CIs). All statistical analyses were performed using SPSS software (version 28.0; IBM, Armonk, NY, USA).

## Results

### Patient characteristics

In all, 240 people from 11 primary care centers were screened for participating in this study. Of those, 31 were found ineligible due to an age below 60 years. Of the remaining 209, 12 people refused to participate and one did not complete the questionnaire resulting in 196 participants (response rate 93.8%).

The median age of participants was 68.6 years (interquartile range [IQR] 64.4–72.2), and the majority was female (73%). Most were prescribed one to five regular medicines. This included metformin in 84.2%, blood pressure-lowering medicines in 67.9%, and lipid-lowering medicines in 28.1% (in all cases statins). About 33% of the participants used CAM, often biologically-based such as herbal therapy (Table 1).

**Table 1:** Patient characteristics (*n* = 196).

**Age (years), median (IQR)**	68.6	(64.4–72.2)
**Sex, n (%)**		
Female	143	(73.0)
Male	52	(26.5)
Unknown	1	(0.5)
**Number of medicines, n (%)**		
1–5	169	(86.2)
6–10	15	(7.7)
Missing	12	(6.1)
**Cardiometabolic medicines, n (%)**		
Metformin	166	(84.7)
Sulfonylurea	105	(53.6)
Acarbose	27	(13.8)
Insulin	8	(4.1)
Blood pressure-lowering medicines	133	(67.9)
Lipid lowering-medicines (only statins)	55	(28.1)
**Glucose lowering-medicines, n (%)**		
Using 1 non-insulin GL medicine	91	(46.4)
Using 2 non-insulin GL medicine	89	(45.4)
Using 3 non-insulin GL medicine	8	(4.1)
Using insulin with/without other	8	(4.1)
**Combinations of medicines used, n (%)**		
GL medicine	50	(25.5)
GL + BP medicine	91	(46.4)
GL + LL medicine	13	(6.6)
G L + BP + LL medicine	42	(21.4)
**Education level, n (%)**		
Primary school/no school	56	(28.6)
Junior high school	33	(16.8)
Senior high school	64	(32.7)
University degree	43	(21.9)
**Using CAM, n (%)**	64	(32.7)
**Type of CAM, n (%)**		
Skill-based therapy	23	(11.7)
Biologically-based therapy	56	(28.6)
Supernatural therapy	2	(1.0)
Spiritual therapy	1	(0.5)
**Number of CAM, n (%)**		
Using 1 CAM	48	(24.5)
Using 2 CAM	14	(7.1)
Using 3 CAM	2	(1.0)
Abbreviations: *IQR* interquartile range, *GL* Glucose-lowering medicines, *BP* Blood pressure-lowering medicines, *LL* Lipid-lowering medicines, *CAM* Complementary/alternative medicines		

### Attitudes towards deprescribing in general

In general, most participants were satisfied with their current medicines (94%). Still, 69 and 67% of participants were willing to stop one of more their regular medicines if this was recommended by their GP or their specialist, respectively, whereas only 41% would be willing if their pharmacist would say it was possible (Table 2). This difference in willingness to stop medication was dependent on the type of HCP who made the recommendation (*p*-value < 0.001).

**Table 2:** Patients’ responses to global statements

	Strongly disagree and disagree (n, %)	Unsure (n, %)	Strongly agree and agree (n, %)
**Satisfaction with medicines**^**a**^ (*n* = 196)	9 (4.6)	3 (1.5)	184 (93.9)
**Willingness to stop if** ^**b,c**^			
General practitioner proposal (n = 196)	55 (28.0)	6 (3.1)	135 (68.9)
Specialist proposal (n = 183)	53 (29.0)	7 (3.8)	123 (67.2)
Pharmacist proposal (*n* = 195)	105 (53.9)	11 (5.6)	79 (40.5)

^a^ Overall, I’m satisfied with my current medicines

^b^ If my [general practitioner] / [specialist] / [pharmacist] said it was possible, I would be willing to stop one or more of my regular medicines’.

^c^ Chi-square comparing willingness across 3 healthcare professional groups: 40.7, *p*-value < 0.001; Chi-square comparing willingness between general practitioner and specialist: 1.2, *p*-value 0.54)

**Table 3:** Patients’ responses and sum scores for the Patients’ Attitudes Towards Deprescribing

Item	Strongly disagree and disagree (%)	Unsure (%)	Strongly agree and agree (%)
**Burden**, Mean (SD): 2.27 (0.78)			
I feel that I am taking a large number of medicines (n = 196)	64.3	0.5	35.2
Taking my medicines every day is very inconvenient (n = 195)	80.0	1.5	18.5
I spend a lot of money on my medicines (*n* = 196)	94.9	1.0	4.1
Sometimes I think I take too many medicines (n = 196)	66.3	0.5	33.2
I feel that my medicines are a burden to me (*n* = 193)	82.9	1.0	16.1
**Appropriateness,** Mean (SD): 3.71 (0.61)			
I would like to try stopping one of my medicines to see how I feel without it (*n* = 194)	78.4	1.0	20.6
I would like my doctor to reduce the dose of one or more of my medicines (*n* = 196)	50.5	2.6	46.9
I feel that I may be taking one or more medicines that I no longer need (*n* = 196)	82.6	2.6	14.8
I believe one or more of my medicines may be currently giving me side effects (*n* = 195)	87.2	3.1	9.7
I think one or more of my medicines may not be working (*n* = 195)	87.7	3.1	9.2
**Concerns about stopping**, Mean (SD): 2.60 (0.62)			
I have had a bad experience when stopping a medicine before (*n* = 194)	83.0	2.6	14.4
I would be reluctant to stop a medicine that I had been taking for a long time (*n* = 195)	41.5	2.1	56.4
If one of my medicines was stopped I would be worried about missing out on future benefits (*n* = 195)	21.5	2.1	76.4
I get stressed whenever changes are made to my medicines (*n* = 196)	81.2	2.0	16.8
If my doctor recommended stopping a medicine I would feel that he/she was giving up on me (*n* = 196)	90.8	2.6	6.6
**Involvement**, Mean (SD): 3.96 (0.61)			
I like to be involved in making decisions about my medicines with my doctors (*n* = 195)	3.1	0.5	96.4
I have a good understanding of the reasons I was prescribed each of my medicines (*n* = 195)	3.6	0.5	95.9
I like to know as much as possible about my medicines (*n* = 195)	19.5	2.0	78.5
I always ask my doctor, pharmacist or other health care professional if there is something I don’t understand about my medicines (*n* = 195)	23.6	2.6	73.8
I know exactly what medicines I am currently taking, and/or I keep an up to date list of my medicines (*n* = 195)	6.7	0.5	92.8

Around a third of participants felt that they were taking a large number of medicines or were taking too many medicines (Table 3). Few participants were burdened by inconvenience or costs of the medicines. More than 80% of participants believed that their medicines were appropriate in terms of giving benefits and no harms. On the other hand, almost half of them would like their doctor to reduce the dose of one or more of their medicines. Some opposing attitudes were observed related to the patients’ concerns about stopping medicines. Although few participants (6.6%) would feel that their doctor was giving up on them if he/she recommended to stop a medicine, many would be worried about missing out on future benefits if one of their medicines was stopped (76.4%). Agreement with all ‘involvement’ statements was high (78.5–96.4%), indicating that most participants believed they had good knowledge of their medication and would like to be involved in medication decisions.

### Attitudes towards deprescribing specific cardiometabolic medicines

**Fig. 1:**
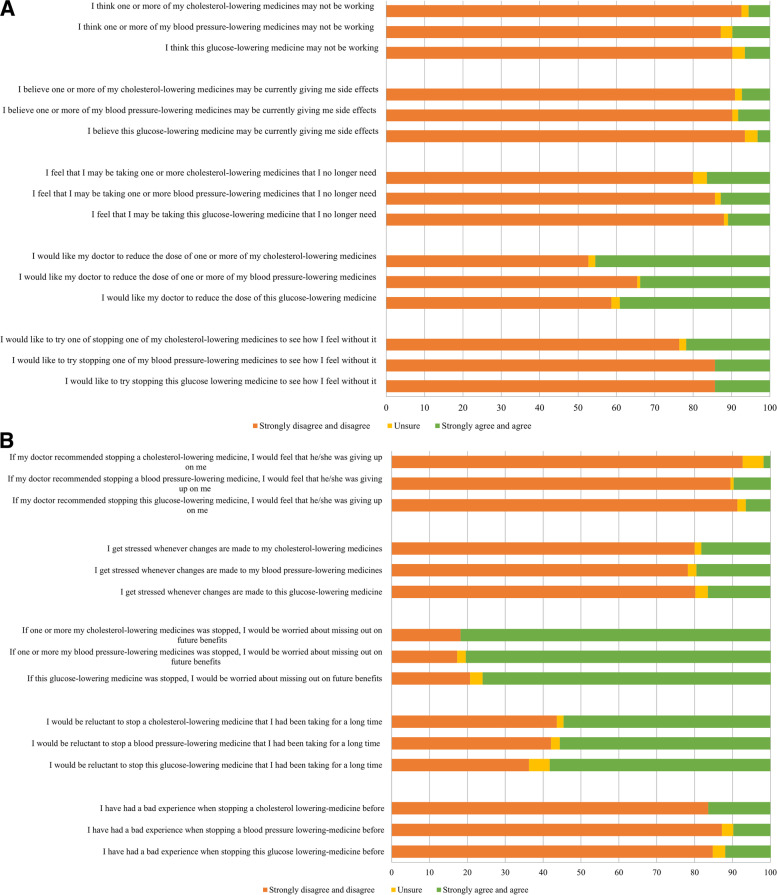
(A) Patients’ responses to appropriateness statements for specific medicines (B) Patients’ responses to concerns statements for specific medicines

Few participants using sulfonylurea or blood-pressure lowering medicines would like to try stopping such medication to see how they would feel without it (14.3%), whereas this was somewhat higher (21.8%) for lipid-lowering medicines (Fig. 1A). At least one third would like their doctor to reduce the dose of their sulfonylurea (39.1%), blood-pressure lowering medicine (33.8%), or lipid-lowering medicine (45.5%) (Fig. 1A, Additional file 3). Most participants would be worried about missing out on future benefits if their sulfonylurea (76.1%), blood pressure-lowering medicines (80.5%) or lipid-lowering medicines (81.8%) were stopped (Fig. 1B., Additional file 3).

### Patient-related factors associated with willingness

In the univariate models, using more than one glucose-lowering medicine, using lipid lowering-medicines, perceived burden of medicines, appropriateness of medicines, and concerns about stopping sum scores were associated with willingness to stop if proposed by the GP or specialist (Additional files 4 and 5). Of note, the strongest associations were seen for the appropriateness statement ‘I would like my doctor to reduce the dose of one or more of my medicines’, the concern statement ‘I would be reluctant to stop a medicine that I had been taking for a long time’, and the burden statement ‘I feel that I am taking a large number of medicines’ (Additional file 6). When proposed by a pharmacist, patients with very little education were more willing to stop than patients with more education (Additional file 4).

In the multiple regression models, higher perceived burden of medicines and lower concerns about stopping were associated with a higher willingness to stop, if this was proposed by the GP or specialist. In addition, a lower perceived appropriateness of medicines was associated with more willingness to stop if the GP proposed this. On the other hand, using more than one glucose-lowering medicine was associated with less willingness to stop their medicines if proposed by the GP. If the proposal to stop the medicines was given by the pharmacist, participants with the lowest level of education were more willing to stop the medicines compared to participants with higher levels of education (Table 4).

**Table 4:** Patient-related factors associated with willingness to stop medicines if the GP, specialist or pharmacist proposes this (multiple regression analyses)

	Willingness if GP proposes^a^ (***n*** = 185)			Willingness if specialist proposes^a^ (***n*** = 173)			Willingness if pharmacist proposes^a^ (***n*** = 183)		
	p-value	aOR	95% CI	p-value	aOR	95% CI	p-value	aOR	95% CI
Appropriateness sum score (reversed)	**0.03**	**0.45**	**0.22–0.93**	0.07	0.54	0.27–1.06	–	–	–
Burden sum score	**0.03**	**1.86**	**1.06–3.28**	**0.046**	**1.72**	**1.01–2.91**	–	–	–
Concerns sum score	**< 0.001**	**0.26**	**0.13–0.51**	**< 0.01**	**0.42**	**0.23–0.76**	–	–	–
Number of medicines	–	–	–	–	–	–	0.21	0.87	0.71–1.08
Using > 1 glucose-lowering medicine^b^	**0.02**	**0.43**	**0.21–0.87**	0.07	0.53	0.27–1.04	–	–	–
Using lipid-lowering medicine^c^	0.11	2.00	0.86–4.67	–	–	–	–	–	–
Education level^d^									
Junior high school	–	–	–	–	–	–	**0.02**	**0.31**	**0.12–0.79**
Senior high school	–	–	–	–	–	–	**0.03**	**0.44**	**0.20–0.93**
University degree	–	–	–	–	–	–	**0.03**	**0.40**	**0.17–0.94**

^a^ Willingness scores are binary categories: (1) strongly agree or agree versus, (2) unsure, disagree, or strongly disagree

^b^ Using one glucose-lowering medicine as reference

^c^ Using no lipid-lowering medicine as reference

^d^ Primary school or no school as reference

Abbreviations: *GP* General practitioner, *aOR* adjusted Odds ratio, *CI* Confidence interval

## Discussion

### Principal findings and comparison with literature

About two-thirds of Indonesian older adults with T2D were willing to stop one or more medicines in general if their GP or specialist said it was possible. This willingness was significantly lower when the pharmacist would propose deprescribing. There was, however, variation in how participants perceived appropriateness and concerns about stopping specific cardiometabolic medicines. Furthermore, participants perceiving more burden of their medicines and having less concerns about stopping were more willing to stop their medicines if their doctor would propose this. Surprisingly, if such a proposal would come from the pharmacists, participants with very limited education were more willing to stop in comparison to those with higher education.

Our findings about the general satisfaction with the medications and willingness to stop medicines are in line with previous studies using the rPATD questionnaire in other Asian countries, where around 80% of older patients were satisfied with their current medications and about two-thirds were willing to stop one or more of their regular medicines if the doctor said it was possible [20, 21]. This willingness is, however, much lower than the 88% willingness observed among primary care patients with T2D in The Netherlands [11]. In general, patients from LMICs, including also those from Indonesia in our study, appear less willing to stop medication compared to high-income countries [9]. Whether this is due to differences in the healthcare system or culture is not clear. Contrary to our expectation, we did not observe any relationship between willingness and the use of CAM. Another study showed that it is common for T2D patients in Indonesia to use CAM in addition to their regular diabetes treatment [22]. This may suggest that they believe that both are needed, without a clear preference of using CAM over regular medicines.

The observed difference between The Netherlands and Indonesia may partly be due to differences in the patient population. We included patients of 60 years and older because life expectancy is lower in Indonesia as compared to countries like the Netherlands. Possibly, not all the participants in our study were people who are eligible for deprescribing, since patients with lower ages can still benefit from continuing their current treatment. On the other hand, we observed no association between age and willingness within our study population. Of note, almost half or our participants used only one glucose-lowering medicine, less than 60% used SU or insulin, and just over 20% used a combination of glucose-lowering, blood pressure-lowering and lipid-lowering medication. This suggests a relatively low need for deprescribing of such drugs. Nonetheless, around a third of our participants did feel burdened by a large number of drugs, which is similar to the findings in the Netherlands [11]. As expected, people perceiving more burden were more willing to stop one or more of the medicines if proposed by their doctor. Surprisingly, people using more than one glucose-lowering medicine were less willing to stop their medicines if proposed by their doctor, whereas these people would be more likely the ones who are eligible for deprescribing. This association became non-significant in the multiple regression model for the specialist. It might be that participants who require more intensive glucose-lowering treatment would trust the specialist more than the GP for reducing such medication.

Looking at the attitudes towards deprescribing, we observed the expected associations between perceived burden of medicines, appropriateness of medicines, and concerns about stopping medicines with a patient’s willingness to stop if a doctor would propose this. However, we observed no such associations with willingness if the pharmacist would say that stopping was possible. This suggests that accepting such proposals from the pharmacist is not related to the patients’ attitudes towards deprescribing. Instead, accepting such proposals appeared mostly related to a patient’s educational level. In general, we identified that the pharmacist appeared to be less trusted to propose changes in medication in Indonesia, particularly among more educated people. Although pharmacists are allowed to give such recommendations to a GP or a specialist, it might be considered inappropriate to make such a suggestion directly to a patient [22, 23]. A previous study in Indonesia about patients’ perceptions of the importance of pharmacist service that can improve medication adherence showed that most patients preferred regular face-to-face consultation over other more intensive pharmacist services, like medication reviews [24]. Educational level appeared to influence the patients’ preference for specific services, with people with primary education being more in favour of medication reviews conducted by pharmacists [24]. Of note, a quarter of the patients had never received any of the pharmacist services, indicating that they may see the pharmacist mostly as someone dispensing and preparing the medications. This may lead to not preferring any other pharmacist services than the short face-to-face consultation when collecting the medication [25]. In a study conducted in the USA, one in five patients indicated never communicating with the pharmacist, but when such communication was perceived effective it was associated with a higher willingness to accept deprescribing [14]. A study in Singapore found that only half of the patients felt comfortable with pharmacists being involved in the deprescribing process in primary care [26], while more than 70% of patients in Croatia had a positive opinion on pharmacists’ involvement in deprescribing [12].. In some high-income countries, there is already involvement of the pharmacist in the deprescribing process, such as taking part in medication reviews that include the option of stopping certain medication, also giving guidance on how to taper and stop specific medicines, and participating in the shared-decision making process [27–29].

Some variation in attitudes related to the appropriateness of medicines and concerns about stopping was seen according to the type of cardiometabolic medicines. In general, it seemed that particularly more of the patients using lipid-lowering medicines would like to try stopping or having their doctor to reduce the dose of this medicine. This may in part be related to a lower perceived need for these medicines and also to differences in perceived disease severity [30]. This is in line with a similar study comparing appropriateness and concerns of cardiometabolic medicines in the Netherlands, where statins were considered less appropriate than blood pressure-lowering medicines and also in comparison to insulin [11]. This indicates that patients with T2D may be more open to stopping statins than their glucose-lowering or blood pressure-lowering medicines.

This study confirmed that there are no clear and consistent associations of patients’ demographics in relation to willingness to stop medicines, as was also found in the previous reviews [8–10]. Sex and age do not seem relevant, but education and number of drugs used may influence willingness although not always too the same extent [8–10]. In our study, the total number of drugs used appeared to be low, which may explain the lack of association with willingness. It was suggested before that the influence of total number of medication might only be seen among populations that use more drugs [9].

### Strengths and limitations

A clear strength of our study is the high response rate. Surprisingly, there was a relatively high number of women participants. A previous survey study among T2D patients in primary care centers in several big cities in Indonesia showed a similar pattern with more women included [31]. It was speculated that this could be because women in Indonesia are more obedient to getting their T2D check-ups regularly than men. Participants were recruited from 11 primary care centers, reflecting a broad city population in Indonesia. However, the results may not reflect the general responses of the overall Indonesian population since patient attitudes may be different in more remote areas. We did not adjust for clustering but observed no clear differences in willingness across the centers (data not shown). Furthermore, the inclusion of participants was somewhat hampered due to COVID restrictions in Indonesia during the study period. When looking at attitudes towards deprescribing of specific cardiometabolic medicines, we did not formally tested for differences since our design would result in a mix of within and between patient comparisons. Finally, all patient-related factors were self reported. Particularly when reporting on the total number of medicines patients use, recall bias and uncertainty about which drugs to include may result in an underestimation of the actual number and type of medicines taken.

### Implications for practice and research

Deprescribing of cardiometabolic medicines is still a new intervention in LMICs. Both patients and HCPs play a role in implementing successful deprescribing. When GPs and specialists want to start deprescribing cardiometabolic medicines among older T2D patients, it is important that they pay attention to concerns of these patients, such as being worried about missing out on future benefits. Also, addressing the perceived appropriateness of specific medicines should be also considered. This appears particularly important for patients using more than one glucose-lowering medicine. Tailoring the deprescribing approach to the individual patient requires talking with the patient about the medication. Shared decision making between patients and HCPs is considered a fundamental part of the deprescribing process [32]. This may require a change in culture in countries like Indonesia and may also take more time investment of HCPs, which can be problematic especially for countries that lack funding for such specific program development.

It seems that many patients in Indonesia are not yet ready for accepting pharmacists to be involved in the deprescribing process. Currently, there is no regulation that gives pharmacists a specific task in this process [22, 23]. A lack of trust from patients but also little existing collaboration of pharmacists with other HCPs may hamper the realization of medication optimization at the primary healthcare level in Indonesia. Pharmacists in Indonesia may also need more training to gain trust. A qualitative study conducted in Iran concluded that to gain trust from the patients and establish an effective relationship with patients, pharmacists need to improve their communication skills and implement the principles of professionalism [33]. In addition, collaboration among HCPs in primary care needs to be enhanced. Developing mutual trustworthiness, initiating a relationship by conducting good communication during the early stages of the relationship, and maintaining high-quality pharmacist contributions have been mentioned as relevant for a successful collaboration [34].

Future research could investigate other patients’ characteristics that might be associated with willingness, such as relationship with the HCPs, frailty, medication adherence, or family support. Furthermore, it would be interesting to conduct research in Indonesia or other LMIC to explore HCP’s opinions regarding deprescribing in T2D patients, as has been done in other countries [27, 28].

## Conclusion

Only two-thirds of older people with T2D in Indonesia were willing to stop one of their medicines if proposed by their GP or specialist, whereas just over 40% were willing to stop if proposed by their pharmacist. The latter was particularly lower among more educated people. Further attention should be given to concerns about stopping among patients using more glucose-lowering medicines, who may be more eligible but were less willing for deprescribing. Finally, CAM use did not seem to impact the willingness to stop regular medicines.

## Supplementary Information

**Additional file 1.** The Checklist for Reporting Of Survey Studies (CROSS)

**Additional file 2.** Patient data collection form, the revised Patient’s Attitudes Towards Deprescribing (rPATD) questionnaire, and the informed consent form in English and in Indonesian (as used in the study)

**Additional file 3.** Patients’ responses to appropriateness and concerns statements for specific medicines

**Additional file 4.** Univariate analyses for associations between patients’ characteristics and willingness

**Additional file 5.** Univariate analyses for associations between sum scores of revised Patient’s Attitudes Towards Deprescribing domains and willingness

**Additional file 6.** Univariate analyses for associations between individual statements of revised Patient’s Attitudes Towards Deprescribing and willingness

## Data Availability

The datasets supporting the conclusions of this article are included within the article and additional files.

## References

[1] Munshi MN, Maguchi M, Segal AR (2012). Treatment of type 2 diabetes in the elderly. Curr Diab Rep.

[2] Draznin B, Aroda VR, Bakris G, Benson G, Brown FM, American Diabetes Association Professional Practice Committee (2022). 13. Older adults: standards of medical Care in Diabetes-2022. Diabetes Care.

[3] Davies MJ, D'Alessio DA, Fradkin J, Kernan WN, Mathieu C, Mingrone G (2018). Management of Hyperglycemia in type 2 diabetes, 2018. A consensus report by the American Diabetes Association (ADA) and the European Association for the Study of diabetes (EASD). Diabetes Care.

[4] Draznin B, Aroda VR, Bakris G, Benson G, Brown FM, American Diabetes Association Professional Practice Committee (2022). 9. Pharmacologic approaches to glycemic treatment: standards of medical Care in Diabetes-2022. Diabetes Care.

[5] Reeve E, Gnjidic D, Long J, Hilmer S (2015). A systematic review of the emerging definition of 'deprescribing' with network analysis: implications for future research and clinical practice. Br J Clin Pharmacol.

[6] Oktora MP, Kerr KP, Hak E, Denig P (2021). Rates, determinants and success of implementing deprescribing in people with type 2 diabetes: a scoping review. Diabet Med.

[7] Reeve E, Thompson W, Farrell B (2017). Deprescribing: a narrative review of the evidence and practical recommendations for recognizing opportunities and taking action. Eur J Intern Med.

[8] Chock YL, Wee YL, Gan SL, Teoh KW, Ng KY, Lee SWH (2021). How willing are patients or their caregivers to Deprescribe: a systematic review and Meta-analysis. J Gen Intern Med.

[9] Oktora MP, Edwina AE, Denig P (2022). Differences in older Patients' attitudes toward Deprescribing at contextual and individual level. Front Public Health.

[10] Weir KR, Ailabouni NJ, Schneider CR, Hilmer SN, Reeve E (2022). Consumer attitudes towards Deprescribing: a systematic review and Meta-analysis. J Gerontol A Biol Sci Med Sci.

[11] Crutzen S, Abou J, Smits SE, Baas G, Hugtenburg JG, Heringa M (2021). Older people's attitudes towards deprescribing cardiometabolic medication. BMC Geriatr.

[12] Buzancic I, Dragovic P, Pejakovic TI, Markulin L, Ortner-Hadziabdic M (2021). Exploring Patients' attitudes toward Deprescribing and their perception of pharmacist involvement in a European country: a cross-sectional study. Patient Prefer Adherence.

[13] Alfian SD, Sukandar H, Arisanti N, Abdulah R (2016). Complementary and alternative medicine use decreases adherence to prescribed medication in diabetes patients. Ann. Trop. Med. Public Health.

[14] Lukacena KM, Keck JW, Freeman PR, Harrington NG, Huffmyer MJ, Moga DC (2022). Patients' attitudes toward deprescribing and their experiences communicating with clinicians and pharmacists. Ther Adv Drug Saf.

[15] Raosoft. Raosoft sample size calculator 2004 [Available from: http://www.raosoft.com/samplesize.html.

[16] Sharma A, Minh Duc NT, Luu Lam Thang T, Nam NH, Ng SJ, Abbas KS (2021). A consensus-based checklist for reporting of survey studies (CROSS). J Gen Intern Med.

[17] Reeve E, Low LF, Shakib S, Hilmer SN (2016). Development and validation of the revised Patients' attitudes towards Deprescribing (rPATD) questionnaire: versions for older adults and caregivers. Drugs Aging.

[18] Wild D, Grove A, Martin M, Eremenco S, McElroy S, Verjee-Lorenz A (2005). Principles of good practice for the translation and cultural adaptation process for patient-reported outcomes (PRO) measures: report of the ISPOR task force for translation and cultural adaptation. Value Health.

[19] Reeve E, Low LF, Hilmer SN (2019). Attitudes of older adults and caregivers in Australia toward Deprescribing. J Am Geriatr Soc.

[20] Kua KP, Saw PS, Lee SWH (2019). Attitudes towards deprescribing among multi-ethnic community-dwelling older patients and caregivers in Malaysia: a cross-sectional questionnaire study. Int J Clin Pharm.

[21] Shrestha S, Giri R, Sapkota HP, Danai SS, Saleem A, Devkota S (2021). Attitudes of ambulatory care older Nepalese patients towards deprescribing and predictors of their willingness to deprescribe. Ther Adv Drug Saf.

[22] Sari Y, Anam A, Sumeru A, Sutrisna E (2021). The knowledge, attitude, practice and predictors of complementary and alternative medicine use among type 2 diabetes mellitus patients in Indonesia. J Integr Med.

[23] Purnama NR, Syamsuddin F, Lestari SB (2009). Pedoman Pemantauan Terapi Obat. Indonesia: Direktorat Bina Farmasi Komunitas dan Klinik, Direktorat Jendral Bina Kefarmasian dan Alat Kesehatan.

[24] Presley B, Groot W, Pavlova M (2021). Pharmacists' and patients' perceptions about the importance of pharmacist services types to improve medication adherence among patients with diabetes in Indonesia. BMC Health Serv Res.

[25] Presley B, Groot W, Widjanarko D, Pavlova M (2021). Preferences for pharmacist services to enhance medication management among people with diabetes in Indonesia: a discrete choice experiment. Patient Educ Couns.

[26] Ng WL, Tan MZW, Koh EYL, Tan NC (2017). Deprescribing: what are the views and factors influencing this concept among patients with chronic diseases in a developed Asian community?. Proc. Singapore Healthc.

[27] Abou J, Crutzen S, Tromp V, Heringa M, Van Marum R, Elders P (2022). Barriers and enablers of healthcare providers to Deprescribe Cardiometabolic medication in older patients: a focus group study. Drugs Aging.

[28] Gerlach N, Michiels-Corsten M, Viniol A, Schleef T, Junius-Walker U, Krause O (2020). Professional roles of general practitioners, community pharmacists and specialist providers in collaborative medication deprescribing - a qualitative study. BMC Fam Pract.

[29] Anderson K, Foster M, Freeman C, Luetsch K, Scott I (2017). Negotiating “unmeasurable harm and benefit”: perspectives of general practitioners and consultant pharmacists on deprescribing in the primary care setting. Qual Health Res.

[30] Long E, Ponder M, Bernard S (2017). Knowledge, attitudes, and beliefs related to hypertension and hyperlipidemia self-management among African-American men living in the southeastern United States. Patient Educ Couns.

[31] Alfian SD, Annisa N, Fajriansyah F, Perwitasari DA, Abdulah R, Hak E (2020). Modifiable factors associated with non-adherence to antihypertensive or Antihyperlipidemic drugs are dissimilar: a multicenter study among patients with diabetes in Indonesia. J Gen Intern Med.

[32] Jansen J, Naganathan V, Carter SM, McLachlan AJ, Nickel B, Irwig L (2016). Too much medicine in older people? Deprescribing through shared decision making. BMJ.

[33] Esmalipour R, Salary P, Shojaei A (2021). Trust-building in the Pharmacist-patient Relationship: a qualitative study. Iran J Pharm Res.

[34] Snyder ME, Zillich AJ, Primack BA, Rice KR, Somma McGivney MA, Pringle JL (2010). Exploring successful community pharmacist-physician collaborative working relationships using mixed methods. Res Social Adm Pharm.

