# Supplementary material for: Attitudes towards deprescribing and patient-related factors associated with willingness to stop medication among older patients with type 2 diabetes (T2D) in Indonesia: a cross-sectional survey study
Source: BMC Geriatr. 2023 Jan 12;23:21. doi: 10.1186/s12877-022-03718-9 (PMC9835373; doi:10.1186/s12877-022-03718-9)
Supplement: Supplementary file 2 — Additional file 2. Patient data collection form, the revised Patient’s Attitudes Towards Deprescribing (rPATD) questionnaire, and the informed consent form in English and in Indonesian (as used in the study) [file 12877_2022_3718_MOESM2_ESM.pdf]

Additional file 2

Patient data collection form, the revised Patient's Attitudes Towards Deprescribing (rPATD) questionnaire, and the informed consent form in English

Participant code: ____ Visit date (dd/mm/yyyy): __ /__ /____

Investigation into the ideas of older adults about medicines

Location of data retrieval: __________

Date of birth (dd/mm/yyyy): _ _ /_ _ /_ _ _ _

Sex: ☐ Male ☐ Female

The current list of regular medicines taken (excluding traditional/alternative medicine): ___

Diabetes medication and/or cardiovascular medicines that you got:

- ☐ Biguanide (e.g metformin)
- ☐ Sulfonylurea (e.g glibenclamid, glyburide, glipizide, glimepiride, or others)
- ☐ Blood pressure lowering medicines (e.g. captopril, amlodipine, nifedipine, furosemide, HCT, atenolol, bisoprolol, sartans, or others)
- ☐ Cholesterol lowering medicines (e.g simvastatin, atorvastatin, lovastatin, or others)

Education:

- ☐ No school/Primary school
- ☐ Junior high school
- ☐ Senior high school
- ☐ University degree

Do you take any complementary and alternative medicine at this moment?

- ☐ NO (continue to the next page)
- ☐ YES (continue to the next question)

Which complementary and alternative medicine that you took?

- ☐ Skill-based therapy (e.g massage, reflexology, acupuncture, acupressure, or other similar treatments)
- ☐ Biologically based therapy (e.g herbal medicine, shinse, aromatherapy, homeopathy or other similar treatments)
- ☐ Supernatural therapy (e.g supernatural power, psychics, shamans, or other similar treatments)
- ☐ Spiritual therapy (e.g traditional healers with an approach of Islam, Christianity, Catholicism, Hinduism, or Buddhism)

Investigation into the ideas of older adults about medicines

There are no right or wrong answers, please tick the box to indicate how strongly you agree with each of the following statements. If there are any questions that you cannot answer, or feel that it doesn't apply to you, please skip it and move to the next question.

For the questions that ask about your doctor, please think of the doctor that prescribes the most (if not all) of your medicines.

	Strongly Agree	Agree	Unsure	Disagree	Strongly Disagree	Not Applicable
Overall, I am satisfied with my current medicines						
I like to be involved in making decisions about my medicines with my doctors						
I have a good understanding of the reasons I was prescribed each of my medicines						
I like to know as much as possible about my medicines						
I always ask my doctor, pharmacist or other health care professional if there is something I don't understand about my medicines						
I know exactly what medicines I am currently taking, and/or I keep an up to date list of my medicines						
If my primary care doctor said it was possible, I would be willing to stop one or more of my regular medicines						
If my specialist said it was possible, I would be willing to stop one or more of my regular medicines						
If my pharmacist said it was possible, I would be willing to stop one or more of my regular medicines						
I feel that I am taking a large number of medicines						
Taking my medicines every day is very inconvenient						
I spend a lot of money on my medicines						

Sometimes I think I take too many medicines					
I feel that my medicines are a burden to me					
I would like to try stopping one of my medicines to see how I feel without it					
I would like my doctor to reduce the dose of one or more of my medicines					
I feel that I may be taking one or more medicines that I no longer need					
I believe one or more of my medicines may be currently giving me side effects					
I think one or more of my medicines may not be working					
I have had a bad experience when stopping a medicine before					
I would be reluctant to stop a medicine that I had been taking for a long time					
If one of my medicines was stopped I would be worried about missing out on future benefits					
I get stressed whenever changes are made to my medicines					
If my doctor recommended stopping a medicine I would feel that he/she was giving up on me					

Specific questions about my blood-pressure lowering medicines*: ………………………

*remark for research assistant: note down and explain to which drugs this refers

	Strongly Agree	Agree	Unsure	Disagree	Strongly Disagree
I would like to try stopping one of my blood pressure-lowering medicines to see how I feel without it					
I would like my doctor to reduce the dose of one or more of my blood pressure-lowering medicines					
I feel that I may be taking one or more blood pressure lowering medicines that I no longer need					
I believe one or more of my blood pressure-lowering medicines may be currently giving me side effects					
I think one or more of my blood pressure-lowering medicines may not be working					
I have had a bad experience when stopping a blood pressure-lowering medicine before					
I would be reluctant to stop a blood pressure-lowering medicine that I had been taking for a long time					
If one or more my blood pressure-lowering medicines was stopped, I would be worried about missing out on future benefits					
I get stressed whenever changes are made to my blood pressure-lowering medicines					
If my doctor recommended stopping a blood pressure-lowering medicine, I would feel that he/she was giving up on me					

Specific questions about my cholesterol lowering medicines*: ……………………….

*remark for research assistant: note down and explain to which drugs this refers

	Strongly Agree	Agree	Unsure	Disagree	Strongly Disagree
I would like to try stopping one of my cholesterol lowering-medicines to see how I feel without it					
I would like my doctor to reduce the dose of one or more of my cholesterol-lowering medicines					
I feel that I may be taking one or more cholesterol-lowering medicines that I no longer need					
I believe one or more of my cholesterol-lowering medicines may be currently giving me side effects					
I think one or more of my cholesterol-lowering medicines may not be working					
I have had a bad experience when stopping a cholesterol-lowering medicine before					
I would be reluctant to stop a cholesterol lowering-medicine that I had been taking for a long time					
If one or more my cholesterol-lowering medicines was stopped, I would be worried about missing out on future benefits					
I get stressed whenever changes are made to my cholesterol-lowering medicines					
If my doctor recommended stopping a cholesterol-lowering medicine, I would feel that he/she was giving up on me					

Specific questions about a specific glucose lowering-medicine*: ……………………….

*remark for research assistant: note down and explain this refers only to the sulfonylurea the patient is using (glibenclamide, glimepiride, gliclazide, gliquidone)

	Strongly Agree	Agree	Unsure	Disagree	Strongly Disagree
I would like to try stopping this glucose lowering medicine to see how I feel without it					
I would like my doctor to reduce the dose of this glucose lowering medicine					
I feel that I may be taking this glucose lowering medicine that I no longer need					
I believe this glucose lowering medicine may be currently giving me side effects					
I think this glucose lowering medicine may not be working					
I have had a bad experience when stopping this glucose lowering medicine before					
I would be reluctant to stop this glucose lowering medicine that I had been taking for a long time					
If this glucose lowering medicine was stopped, I would be worried about missing out on future benefits					
I get stressed whenever changes are made to this glucose lowering medicine					
If my doctor recommended stopping this glucose lowering medicine, I would feel that he/she was giving up on me					

Thank you for completing the questionnaire.

For non-commercial use only. Do not adapt or translate without permission.

This is a translated and adapted version of original revised Patients' Attitudes Towards Deprescribing (rPATD) Questionnaire. The original questionnaire publication is available at Springer via http://dx.doi.org/10.1007/s40266-016-0410-1 (Reeve, E., Low, L. F., Shakib, S., & Hilmer, S. N. (2016). Development and Validation of the Revised Patients' Attitudes Towards Deprescribing (rPATD) Questionnaire: Versions for Older Adults and Caregivers. *Drugs & Aging*, *33*(12), 913-928)

PARTICIPANT INFORMATION SHEET

Attitudes towards deprescribing medication among older people with Type 2 Diabetes (T2D) in Indonesia

This study is conducted by Cindra Tri Yuniar (Lecturer at School of Pharmacy, Institut Teknologi Bandung), and Monika Oktora (PhD student), under daily supervision of Lia Amalia, PhD (Senior lecturer, Institut Teknologi Bandung) and Rizky Abdulah, PhD (Universitas Padjadjaran, Indonesia). Researchers are conducting a research to examine the ideas of patients with type 2 diabetes (T2D) about their medicines. The researchers ask your willingness to participate in this study, your participation in this research is voluntary, so you can decide to participate or not.

Research Background and Aims:

Diabetes guidelines have shifted to a more personalized approach, in which treatment can be deintensified, especially in older patients. It is acknowledged that the process of therapy optimization in older patients is being introduced in diabetes care. This process includes of reducing, stopping medicines, or switching to other (lighter) medicines to improve the outcome of therapy and reduce the side effects of therapy. This study aims to examine the process from the patient's point of view, particularly the attitudes and experiences of T2D patients towards the possibility of such changes in their treatment.

Who is invited to take part?

We are interested to include participants who use medicines for T2D and are aged 60 years and above.

Procedures:

1. You will get an explanation about this study, the aim, and the instructions to fill the questionnaire from the research assistance.
2. If you are willing to participate, you are asked to sign the informed consent form and will get the questionnaire to be filled.
3. Research assistance will guide you during the process, and you can ask them if there is question you did not understand.
4. Completing the questionnaire will take approximately 10-15 minutes of your time, while you are waiting for your medication.

Risk or inconvenience:

There is no risk from participating in this study.

Benefit (directly to the participants or to general population):

This study does not directly benefit to the respondents. However, it will be useful in the long term, namely in providing information about optimal therapy in T2D patients.

Confidentiality:

All of the information you provide will be treated confidentially. Your responses and clinical data will not be identifiable to your person. We do not collect your name or address.

Estimated number of participants to include:

Participants with T2D, aged 60 years and older. A sample size between 195 to 385 participants will provide a margin of error of 7% to 5%

Voluntary study

Your participation in this study is voluntary, and you are allowed to stop at any time without completion of the questionnaire.

Time period

Participants will be involved one time in this study to fill the questionnaire (time period November 2021 to March 2022.

Participants can be excluded or withdrawn from this study

You are free to refuse to participate in this study, and you can change your mind anytime. If you do not want to follow the procedures above, you will not be included in this study.

Incentive and compensation:

You will get a souvenir: mask and hand sanitizer, as a compensation of your participation.

Questions:

If you have questions or concerns about this study that you think I can help you with, please feel free to contact me on: Cindra Tri Yuniar, M.Si

Sekolah Farmasi, Institut Teknologi Bandung
Jl. Ganesha 10 40132 Bandung
Phone: 022-xxxxxxx
Mobilephone: +62 xxx-xxxx-xxxx

PARTICIPANT CONSENT FORM

I have read and understood about the aim, benefit, and risk of this study. I have given opportunity to discuss the information and my involvement in the study. I understand that I am free to withdraw my participation from this study at any time I wish, without consequences, and without giving a reason. Therefore, I **agree/disagree**[*)] to participate in the study *Attitudes towards deprescribing medication among older people with Type 2 Diabetes (T2D) in Indonesia*

I understand that being in this study is voluntary, without any pressure from anyone. I agree that the research data gathered from this study may be published in a form that does not identify me in any way.

I agree:

Yes/No[*)]

	Date	Signature
Name:	_ _/_ _/ _ _ _ _	

*) Cross the unnecessary ones.

Patient data collection form, the translated version of revised Patient's Attitudes Towards Deprescribing (rPATD) questionnaire, and the informed consent form in Indonesian

Perhatian sebelum mengisi kuesioner: Kuesioner ini hanya untuk meminta pendapat dan pandangan pasien dalam keyakinannya terhadap obat-obatan yang diterimanya. Pengobatan yang diterima pasien saat ini sudah merupakan pengobatan yang tepat

Kode pasien: ____ Tanggal pengumpulan data (tanggal/bulan/tahun): _ _ /_ _ /_ _

Investigasi Keyakinan Orang lanjut usia terhadap Obat-Obatan

Lokasi pengambilan data: ________________

Tanggal lahir (tanggal/bulan/tahun): _ _ / _ _ / _ _ _ _

Jenis kelamin: ☐ Laki-laki ☐ Perempuan

Jumlah obat-obatan yang Anda gunakan saat ini (tidak termasuk obat tradisional): ____

Obat diabetes dan/atau kardiovaskular yang Anda gunakan (bisa lebih dari satu):

- ☐ Biguanida (contoh: metformin)
- ☐ Sulfonilurea (contoh: glibenklamid, glipizid, glimepirid, glikazid, atau lainnya)
- ☐ Obat antihipertensi (contoh: captopril, amlodipin, nifedipin, furosemid, HCT, atenolol, bisoprolol, golongan sartan, atau lainnya)
- ☐ Statin (simvastatin, atorvastatin, lovastatin, atau lainnya)

Jenjang pendidikan terakhir:

- ☐ Sekolah Dasar (SD)/tidak sekolah
- ☐ Sekolah Menengah Pertama (SMP)
- ☐ Sekolah Menengah Umum (SMA)
- ☐ Vokasi (D1-D2-D3)/Universitas (S1-S2-S3)

Apakah Anda menggunakan pengobatan tradisional saat ini?

- ☐ TIDAK (lanjut ke halaman berikutnya)
- ☐ YA (lanjut ke pertanyaan di bawah)

Pengobatan tradisional apa yang Anda apa gunakan? (bisa lebih dari satu)

- Pengobatan tradisional keterampilan (contoh: pijat, refleksi, akupuntur, akupresur, chiropractor, dan pengobatan lain yang sejenis)
- Pengobatan tradisional ramuan (contoh: jamu, shinse, aromaterapi, homeopathy, dan pengobatan lain yang sejenis)
- Pengobatan tradisional supranatural (contoh: tenaga dalam, paranormal, dukun dan pemgobatan lain yang sejenis)
- Pengobatan tradisional dengan pendekatan agama (contoh: pengobat tradisional dengan pendekatan agama Islam, Kristen, Katolik, Hindu, atau Budha)

Investigasi Keyakinan Orang Lanjut Usia terhadap Obat-Obatan

Tidak ada jawaban benar atau salah, mohon berikan tanda centang pada kotak yang tersedia untuk menunjukkan seberapa besar Anda setuju dengan masing-masing pernyataan berikut. Jika ada pertanyaan yang tidak dapat Anda jawab, atau membingungkan, bisa ditanyakan lebih lanjut kepada asisten peneliti. Jika Anda merasa pertanyaan tersebut tidak berlaku bagi Anda, lewati saja dan lanjutkan ke pertanyaan berikutnya.

Untuk pertanyaan yang berkaitan dengan dokter Anda, mohon pikirkan tentang dokter yang paling sering (atau selalu) memberikan resep obat kepada Anda.

No		Sangat setuju	Setuju	Ragu-ragu	Tidak setuju	Sangat tidak setuju	Tidak berlaku
1	Secara keseluruhan, saya puas dengan obat-obatan saya saat ini.						
2	Saya senang jika dokter melibatkan saya dalam pengambilan keputusan tentang obat-obatan saya						
3	Saya memahami dengan baik apa alasan dokter meresepkan setiap obat untuk saya						
4	Saya ingin mengetahui sebanyak mungkin tentang obat-obatan saya.						
5	Saya selalu bertanya kepada dokter, apoteker, atau petugas kesehatan profesional lainnya jika ada sesuatu yang tidak saya mengerti tentang obat-obatan saya.						
6	Saya tahu persis obat apa yang saya gunakan saat ini, dan/atau saya mengikuti perubahan daftar obat-obatan saya.						
7	Jika dokter memperbolehkan, saya bersedia menghentikan satu atau lebih dari obat-obatan rutin saya.						
8	Jika dokter spesialis memperbolehkan, saya bersedia menghentikan satu atau lebih dari obat-obatan rutin saya.						
9	Jika apoteker memperbolehkan, saya bersedia menghentikan satu atau lebih dari obat-obatan rutin saya.						
10	Saya merasa bahwa saat ini saya mengonsumsi banyak obat.						
11	Minum obat setiap hari merepotkan saya.						

12	Saya menghabiskan banyak uang untuk obat-obatan saya.					
13	Terkadang saya berpikir bahwa saya mengonsumsi terlalu banyak obat.					
14	Saya merasa obat-obatan saya menjadi beban untuk saya.					
15	Saya ingin mencoba menghentikan salah satu dari obat-obatan saya untuk melihat bagaimana rasanya tanpa obat tersebut.					
16	Saya ingin dokter mengurangi dosis dari satu atau lebih obat-obatan saya.					
17	Saya merasa bahwa saya mungkin mengonsumsi satu atau lebih obat-obatan yang tidak lagi saya butuhkan.					
18	Saya yakin bahwa satu atau lebih dari obat-obatan saya mungkin telah memberikan efek samping pada saya.					
19	Saya pikir satu atau lebih dari obat-obatan saya mungkin tidak bekerja dengan baik.					
20	Saya memiliki pengalaman buruk ketika menghentikan obat sebelumnya.					
21	Saya akan enggan untuk menghentikan obat yang sudah saya konsumsi sejak lama.					
22	Jika salah satu obat saya dihentikan, saya khawatir akan kehilangan efeknya di masa yang akan datang.					
23	Saya merasa stres setiap kali ada perubahan pada obat-obatan saya.					
24	Jika dokter merekomendasikan saya untuk berhenti mengonsumsi obat, saya akan merasa bahwa dia telah menyerah pada saya.					

Pertanyaan spesifik mengenai obat penurun tekanan darah tinggi (antihipertensi) (*…………………..…….)

Catatan untuk asisten peneliti, cantumkan nama obat yang digunakan

No		Sangat setuju	Setuju	Ragu-ragu	Tidak setuju	Sangat tidak setuju
AH1	Saya ingin mencoba menghentikan obat penurun tekanan darah tinggi saya untuk melihat bagaimana rasanya tanpa obat tersebut.					
AH2	Saya ingin dokter mengurangi dosis obat penurun tekanan darah tinggi saya.					
AH3	Saya merasa bahwa saya mungkin mengonsumsi satu atau lebih obat-obatan penurun tekanan darah tinggi yang tidak lagi saya butuhkan.					
AH4	Saya yakin obat penurun tekanan darah tinggi saya mungkin telah memberikan efek samping pada saya.					
AH5	Saya pikir obat penurun tekanan darah tinggi saya mungkin tidak bekerja dengan baik.					
AH6	Saya memiliki pengalaman buruk ketika menghentikan obat penurun tekanan darah tinggi sebelumnya.					
AH7	Saya akan enggan untuk menghentikan obat penurun tekanan darah tinggi yang sudah saya konsumsi sejak lama.					
AH8	Jika obat penurun tekanan darah tinggi saya dihentikan, saya khawatir akan kehilangan efeknya di masa yang akan datang.					
AH9	Saya merasa stres setiap kali ada perubahan pada obat penurun tekanan darah tinggi saya.					
AH10	Jika dokter merekomendasikan saya untuk berhenti mengonsumsi obat penurun tekanan darah tinggi, saya akan merasa bahwa dia telah menyerah saya.					

Pertanyaan spesifik mengenai obat penurun kolesterol (*………………..)

*Catatan untuk asisten peneliti, cantumkan nama obat yang digunakan

No		Sangat setuju	Setuju	Ragu-ragu	Tidak setuju	Sangat tidak setuju
AK1	Saya ingin mencoba menghentikan obat penurun kolesterol saya untuk melihat bagaimana rasanya tanpa obat tersebut.					
AK2	Saya ingin dokter mengurangi dosis obat penurun kolesterol saya.					
AK3	Saya merasa bahwa saya mungkin mengonsumsi satu atau lebih obat-obatan penurun kolesterol yang tidak lagi saya butuhkan.					
AK4	Saya yakin obat penurun kolesterol saya mungkin telah memberikan efek samping pada saya.					
AK5	Saya pikir obat penurun kolesterol saya mungkin tidak bekerja dengan baik					
AK6	Saya memiliki pengalaman buruk ketika <u>menghentikan</u> obat penurun kolesterol sebelumnya.					
AK7	Saya akan enggan untuk menghentikan obat penurun kolesterol yang sudah saya konsumsi sejak lama.					
AK8	Jika obat penurun kolesterol saya dihentikan, saya khawatir akan kehilangan efeknya di masa yang akan datang.					
AK9	Saya merasa stres setiap kali ada perubahan pada obat penurun kolesterol saya.					
AK10	Jika dokter merekomendasikan saya untuk berhenti mengonsumsi obat penurun kolesterol, saya akan merasa bahwa dia telah menyerah pada saya.					

Pertanyaan spesifik mengenai obat penurun gula darah (antidiabetes) (*………………..)

*Catatan untuk asisten peneliti, cantumkan nama obat yang digunakan, hanya untuk pengguna sulfonylurea (contoh: glibenklamid, glimepirid, glikazid, glipizid)

No		Sangat setuju	Setuju	Ragu-ragu	Tidak setuju	Sangat tidak setuju
AD1	Saya ingin mencoba menghentikan obat penurun gula darah saya untuk melihat bagaimana rasanya tanpa obat tersebut.					
AD2	Saya ingin dokter mengurangi dosis obat penurun gula darah saya.					
AD3	Saya merasa bahwa saya mungkin mengonsumsi satu atau lebih obat-obatan penurun gula darah yang tidak lagi saya butuhkan.					
AD4	Saya yakin obat penurun gula darah saya mungkin telah memberikan efek samping pada saya.					
AD5	Saya pikir obat penurun gula darah saya mungkin tidak bekerja dengan baik.					
AD6	Saya memiliki pengalaman buruk ketika menghentikan obat penurun gula darah sebelumnya.					
AD7	Saya akan enggan untuk menghentikan obat penurun gula darah yang sudah saya konsumsi sejak lama.					
AD8	Jika obat penurun gula darah saya dihentikan, saya khawatir akan kehilangan efeknya di masa yang akan datang.					
AD9	Saya merasa stres setiap kali ada perubahan pada obat penurun gula darah saya.					
AD10	Jika dokter merekomendasikan saya untuk berhenti mengonsumsi obat penurun gula darah, saya akan merasa bahwa dia telah menyerah pada saya.					

Terima kasih telah mengisi kuesioner.

Hanya dipergunakan untuk keperluan non-komersial saja.

Dilarang mengadaptasi dan menerjemahkan tanpa izin. Kuesioner ini merupakan hasil terjemahan dan adaptasi dari versi asli kuesioener Revised Patients' Attitudes Towards Deprescribing (rPATD). Artikel asli tersedua di Springer via http://dx.doi.org/10.1007/s40266-016-0410-1 (Reeve, E., Low, L. F., Shakib, S., & Hilmer, S. N. (2016). Development and Validation of the Revised Patients' Attitudes Towards Deprescribing (rPATD) Questionnaire: Versions for Older Adults and Caregivers. *Drugs & Aging*, *33*(12), 913-928)
