# Supplementary material for: Attitudes towards deprescribing and patient-related factors associated with willingness to stop medication among older patients with type 2 diabetes (T2D) in Indonesia: a cross-sectional survey study
Source: BMC Geriatr. 2023 Jan 12;23:21. doi: 10.1186/s12877-022-03718-9 (PMC9835373; doi:10.1186/s12877-022-03718-9)
Supplement: Supplementary file 3 — Additional file 3. Patients’ responses to appropriateness and concerns statements for specific medicines [file 12877_2022_3718_MOESM3_ESM.pdf]

Additional file 3. Table patients' responses to appropriateness and concerns statements for specific medicines

Item		Strongly disagree and disagree (%)	Unsure (%)	Strongly agree and agree (%)
**Appropriateness sulfonylurea**				
**Sum score, n = 91, mean (SD) = 3.79 (0.60)**				
SU1	I would like to try stopping this glucose-lowering medicine to see how I feel without it (n = 91)	85.7	0	14.3
SU2	I would like my doctor to reduce the dose of this glucose-lowering medicine (n = 92)	58.7	2.2	39.1
SU3	I feel that I may be taking this glucose-lowering medicine that I no longer need (n = 92)	88.0	1.1	10.9
SU4	I believe this glucose-lowering medicine may be currently giving me side effects (n = 92)	93.5	3.3	3.3
SU5	I think this glucose-lowering medicine may not be working (n = 92)	90.2	3.3	6.5
**Concerns sulfonylurea**				
**Sum score, n = 90, mean (SD) = 2.66 (0.62)**				
SU6	I have had a bad experience when stopping this glucose-lowering medicine before (n = 92)	84.8	3.3	12.0
SU7	I would be reluctant to stop this glucose-lowering medicine (n = 91)	36.3	5.5	58.2
SU8	If this glucose-lowering medicine was stopped, I would be worried about missing out on future benefits (n = 92)	20.7	3.3	76.1
SU9	I get stressed whenever changes are made to this glucose-lowering medicine (n = 91)	80.2	3.3	16.5
SU10	If my doctor recommended stopping this glucose-lowering medicine, I would feel that he/she was giving up on me (n = 92)	91.3	2.2	6.5
**Appropriateness blood pressure-lowering medicines**				
**Sum score, n = 133, mean (SD) = 3.78 (0.59)**				
BP1	I would like to try stopping one of my blood-pressure-lowering medicines to see how I feel without it (n = 133)	85.7	0.0	14.3
BP2	I would like my doctor to reduce the dose of one or more of my blood pressure-lowering medicines (n = 133)	65.4	0.8	33.8
BP3	I feel that I may be taking one or more blood pressure-lowering medicines that I no longer need (n = 133)	85.7	1.5	12.8
BP4	I believe one or more of my blood pressure-lowering medicines may be currently giving me side effects (n = 133)	90.2	1.5	8.3
BP5	I think one or more of my blood pressure-lowering medicines may not be working (n = 133)	87.2	3.0	9.8

**Concerns blood pressure-lowering medicines**				
**n = 133; Mean (SD) = 2.65 (0.58)**				
BP6	I have had a bad experience when stopping a blood pressure-lowering medicine before (n = 133)	87.2	3.0	9.8
BP7	I would be reluctant to stop a blood pressure-lowering medicine that I had been taking for a long time (n = 133)	42.1	2.3	55.6
BP8	If one or more my blood pressure-lowering medicines was stopped, I would be worried about missing out on future benefits (n = 133)	17.3	2.3	80.5
BP9	I get stressed whenever changes are made to my blood pressure-lowering medicines (n = 133)	78.2	2.3	19.5
BP10	If my doctor recommended stopping a blood pressure-lowering medicine, I would feel that he/she was giving up on me (n = 133)	89.5	0.8	9.8
**Appropriateness lipid-lowering medicines**				
**n = 54; Mean (SD) = 3.70 (0.61)**				
LL1	I would like to try stopping one of my cholesterol-lowering medicines to see how I feel without it (n = 55)	76.4	1.8	21.8
LL2	I would like my doctor to reduce the dose of one or more my cholesterol-lowering medicines (n = 55)	52.7	1.8	45.5
LL3	I feel that I may be taking one or more cholesterol-lowering medicines that I no longer need (n = 55)	80.0	3.6	16.4
LL4	I believe one or more of my cholesterol-lowering medicines may be currently giving me side effects (n = 55)	90.9	1.8	7.3
LL 5	I think one or more of my cholesterol-lowering medicines may not be working (n = 54)	92.6	1.9	5.6
**Concerns lipid-lowering medicines**				
**n = 55; Mean (SD) = 2.62 (0.54)**				
LL6	I have had a bad experience when stopping a cholesterol-lowering medicine before (n = 55)	83.6	0	16.4
LL7	I would be reluctant to stop a cholesterol-lowering medicine that I had been taking for a long time (n = 55)	43.6	1.8	54.6
LL8	If one or more my cholesterol-lowering medicines was stopped, I would be worried about missing out on future benefits (n = 55)	18.2	0	81.8
LL9	I get stressed whenever changes are made to my cholesterol-lowering medicines (n = 55)	80.0	1.8	18.2
LL10	If my doctor recommended stopping a cholesterol-lowering medicine, I would feel that he/she was giving up on me (n = 55)	92.7	5.5	1.8
