# Supplementary material for: Attitudes towards deprescribing and patient-related factors associated with willingness to stop medication among older patients with type 2 diabetes (T2D) in Indonesia: a cross-sectional survey study
Source: BMC Geriatr. 2023 Jan 12;23:21. doi: 10.1186/s12877-022-03718-9 (PMC9835373; doi:10.1186/s12877-022-03718-9)
Supplement: Supplementary file 4 — Additional file 4. Univariate analyses for associations between patients’ characteristics and willingness [file 12877_2022_3718_MOESM4_ESM.pdf]

**Additional file 4. Table univariate analyses for associations between patient characteristics and willingness**

| | **Willingness if GP proposes (n = 196)** | | | **Willingness if specialist proposes (n = 183)** | | | **Willingness if pharmacist proposes (n = 195)** | | |
|---|---|---|---|---|---|---|---|---|---|
| | p value | OR | 95 % CI | p value | OR | 95 % CI | p value | OR | 95 % CI |
| **Age group**[a] | | | | | | | | | |
| 65-69 | 0.98 | 0.99 | 0.45-2.19 | 0.68 | 1.19 | 0.53-2.69 | 0.34 | 1.44 | 0.68-3.06 |
| 70-74 | 0.77 | 0.88 | 0.38-2.05 | 0.78 | 0.88 | 0.37-2.09 | 0.65 | 1.21 | 0.54-2.70 |
| > 75 | 0.85 | 1.10 | 0.40-3.06 | 0.70 | 1.23 | 0.44-3.46 | 0.77 | 0.86 | 0.32-2.31 |
| **Sex** (male) | 0.48 | 0.78 | 0.38-1.57 | 0.97 | 1.02 | 0.50-2.04 | 0.79 | 0.91 | 0.48-1.74 |
| **Number of medicines** | 0.79 | 1.03 | 0.84-1.27 | 0.82 | 0.98 | 0.80-1.20 | **0.16** | **0.86** | **0.71-1.06** |
| **Education level**[c] | | | | | | | | | |
| Junior high school | 0.26 | 0.58 | 0.23-1.48 | **0.15** | **0.50** | **0.19-1.27** | **0.01** | **0.27** | **0.11-0.69** |
| Senior high school | 0.45 | 0.73 | 0.33-1.64 | 0.69 | 0.85 | 0.37-1.94 | **0.03** | **0.43** | **0.21-0.90** |
| University degree | 0.29 | 0.62 | 0.26-1.49 | 0.23 | 0.58 | 0.24-1.40 | **0.01** | **0.35** | **0.15-0.80** |
| **Type of medicines** | | | | | | | | | |
| Using >1 glucose-lowering medicine[c] | **0.04** | **0.53** | **0.29-0.98** | **0.13** | **0.62** | **0.33-1.15** | 0.40 | 0.78 | 0.44-1.38 |
| Using blood pressure-lowering medicines[d] | 0.43 | 1.29 | 0.68-2.45 | 0.66 | 1.16 | 0.60-2.23 | 0.88 | 0.96 | 0.52-1.76 |
| Using lipid-lowering medicines[e] | **0.08** | **1.91** | **0.92-3.95** | 0.29 | 1.47 | 0.72-2.99 | 0.82 | 1.08 | 0.57-2.03 |
| **Using CAM**[f] | 0.76 | 1.11 | 0.58-2.12 | 0.49 | 1.28 | 0.64-2.54 | 0.74 | 1.11 | 0.60-2.03 |

Abbreviations: GP: general practitioner, OR: Odds ratio, CI: confidence interval, CAM: complementary and alternative medicines

[a] Age group 60-64 years as reference

[b] Primary school or no school as reference

[c] Using 1 glucose-lowering medicines as reference

[d] Using no blood pressure-lowering medicines as reference

[e] Using no lipid-lowering medicines as reference

[f] Using no CAM as reference
