# Supplementary material for: Attitudes towards deprescribing and patient-related factors associated with willingness to stop medication among older patients with type 2 diabetes (T2D) in Indonesia: a cross-sectional survey study
Source: BMC Geriatr. 2023 Jan 12;23:21. doi: 10.1186/s12877-022-03718-9 (PMC9835373; doi:10.1186/s12877-022-03718-9)
Supplement: Supplementary file 5 — Additional file 5. Univariate analyses for associations between sum scores of revised Patient’s Attitudes Towards Deprescribing domains and willingness [file 12877_2022_3718_MOESM5_ESM.pdf]

Additional file 5. Table univariate analyses for associations between sum scores of revised Patient's Attitudes Towards Deprescribing domains and willingness

	**Willingness if GP proposes**			**Willingness if specialist proposes**			**Willingness if pharmacist proposes**		
	p value	OR	95 % CI	p value	OR	95 % CI	p value	OR	95 % CI
Appropriateness (n = 192)	**0.01**	**0.46**	**0.25-0.84**	**0.01**	**0.45**	**0.24-0.83**	0.58	0.88	0.55-1.41
Burden (n = 192)	**0.02**	**1.63**	**1.07-2.48**	**0.03**	**1.60**	**1.04-2.45**	0.64	1.09	0.75-1.58
Concerns (n = 192)	**<0.001**	**0.42**	**0.24-0.74**	**0.06**	**0.61**	**0.36-1.03**	0.71	1.09	0.69-1.74
Involvement (n = 191)	0.31	1.30	0.78-2.16	0.21	1.40	0.83-2.37	0.87	0.96	0.59-1.56

Abbreviations: GP: general practitioner, OR: Odds ratio, CI: confidence interval, CAM: complementary and alternative medicines
