# Supplementary material for: Attitudes towards deprescribing and patient-related factors associated with willingness to stop medication among older patients with type 2 diabetes (T2D) in Indonesia: a cross-sectional survey study
Source: BMC Geriatr. 2023 Jan 12;23:21. doi: 10.1186/s12877-022-03718-9 (PMC9835373; doi:10.1186/s12877-022-03718-9)
Supplement: Supplementary file 6 — Additional file 6. Univariate analyses for associations between individual statements of revised Patient’s Attitudes Towards Deprescribing and willingness [file 12877_2022_3718_MOESM6_ESM.pdf]

Additional file 6. Table univariate analyses for associations between individual statements of revised Patient's Attitudes Towards Deprescribing and willingness

	Willingness GP*			Willingness Specialist*			Willingness Pharmacist*		
	**p value**	**OR**	**95 % CI**	**p value**	**OR**	**95 % CI**	**p value**	**OR**	**95 % CI**
**Burden**									
I feel that I am taking a large number of medicines (n = 196)	**0.001**	**3.42**	**1.64-7.15**	**0.01**	**2.74**	**1.32-5.67**	0.75	1.10	0.61-2.00
Taking my medicines every day is very inconvenient (n = 195)	**0.003**	**6.32**	**1.85-21.5**	**0.01**	**3.98**	**1.32-11.96**	**0.07**	**1.96**	**0.94-4.10**
I spend a lot of money on my medicines (n = 196)	0.99	> 1000	0.00 - ~	0.24	3.56	0.43-29.62	**0.06**	**4.68**	**0.92-23.8**
Sometimes I think I take too many medicines (n = 196)	**0.01**	**2.64**	**1.28-5.42**	**0.02**	**2.47**	**1.19-5.13**	0.84	1.07	0.58-1.95
I feel that my medicines are a burden to me (n = 193)	**0.02**	**3.66**	**1.22-10.99**	**0.02**	**8.02**	**1.84-35.10**	0.14	1.80	0.83-3.89
**Appropriateness**									
I would like to try stopping one of my medicines to see how I feel without it (n = 194)	**0.02**	3.15	**1.24-7.97**	**0.002**	**5.47**	**1.84-16.26**	0.31	1.44	0.71-2.91
I would like my doctor to reduce the dose of one or more of my medicines (n = 196)	**<0.001**	3.62	**1.86-7.04**	**<0.001**	**3.40**	**1.73-6.67**	0.80	0.93	0.52-1.65
I feel that I may be taking one or more medicines that I no longer need (n = 196)	0.09	2.42	0.877-6.69	0.606	1.26	0.52-3.06	0.92	1.04	0.47-2.32
I believe one or more of my medicines may be currently giving me side effects (n = 195)	0.62	1.31	0.45-3.81	0.892	1.07	0.39-2.98	0.50	1.38	0.53-3.58
I think one or more of my medicines may not be working (n = 195)	0.77	1.17	0.40-3.45	0.415	1.63	0.51-5.21	0.51	0.71	0.25-1.97
**Concerns about stopping**									
I have had a bad experience when stopping a medicine before (n = 194)	0.77	1.14	0.47-2.76	0.96	1.02	0.41-2.52	0.82	1.10	0.49-2.47
I would be reluctant to stop a medicine that I had been taking for a long time (n = 195)	**0.001**	**0.34**	**0.17-0.65**	**0.01**	**0.43**	**0.22-0.83**	0.59	0.85	0.48-1.52
If one of my medicines was stopped I would be worried about missing out on future benefits (n = 195)	**0.01**	**0.32**	**0.13-0.76**	**0.046**	**0.44**	**0.19-0.99**	0.23	0.66	0.34-1.29
I get stressed whenever changes are made to my medicines (n = 196)	0.48	0.75	0.34-1.65	0.52	1.34	0.56-3.23	0.53	1.28	0.60-2.71
If my doctor recommended stopping a medicine I would feel that he/she was giving up on me (n = 196)	0.56	0.71	0.22-2.25	0.65	0.77	0.24-2.45	0.88	0.91	0.29-2.90
**Involvement**									
I like to be involved in making decisions about my medicines with my doctors (n = 195)	0.51	1.68	0.36-7.75	0.38	2.09	0.41-10.67	0.36	0.49	0.11-2.26
I have a good understanding of the reasons I was prescribed each of my medicines (n = 195)	0.70	0.72	0.14-3.69	0.57	1.55	0.34-7.17	0.57	0.66	0.16-2.72

I like to know as much as possible about my medicines (n = 195)	0.96	0.98	0.57-2.05	0.48	1.31	0.62-2.78	0.85	0.94	0.47-1.89
I always ask my doctor, pharmacist or other health care professional if there is something I don’t understand about my medicines (n = 195)	0.42	1.33	0.67-2.61	0.26	1.49	0.74-2.99	0.83	0.93	0.49-1.79
I know exactly what medicines I am currently taking, and/or I keep an up to date list of my medicines (n = 195)	0.82	0.87	0.26-2.89	0.98	1.02	0.29-3.53	0.89	1.08	0.34-3.44
